# Modeling nonequilibrium dynamics of phase transitions at the nanoscale: Application to spin-crossover

**DOI:** 10.1063/1.4985058

**Published:** 2017-06-06

**Authors:** Sang Tae Park, Renske M. van der Veen

**Affiliations:** 1 Physical Biology Center for Ultrafast Science and Technology, Arthur Amos Noyes Laboratory of Chemical Physics, California Institute of Technology, Pasadena, California 91125, USA; 2 Department of Chemistry and Frederick Seitz Materials Research Laboratory, University of Illinois at Urbana-Champaign, Urbana, Illinois 61801, USA

## Abstract

In this article, we present a continuum mechanics based approach for modeling thermally induced single-nanoparticle phase transitions studied in ultrafast electron microscopy. By using coupled differential equations describing heat transfer and the kinetics of the phase transition, we determine the major factors governing the time scales and efficiencies of thermal switching in individual spin-crossover nanoparticles, such as the thermal properties of the (graphite) substrate, the particle thickness, and the interfacial thermal contact conductance between the substrate and the nanoparticle. By comparing the simulated dynamics with the experimental single-particle diffraction time profiles, we demonstrate that the proposed non-equilibrium phase transition model can fully account for the observed switching dynamics.

## I. INTRODUCTION

Controlling and switching the properties and functions of materials at the nanoscale constitute a long-standing goal in a broad range of sciences.^1^ Miniaturization down to nano- and microscopic dimensions (1–1000 nm) is typically required to provide materials of sufficiently small sizes for their use as data storage and optoelectronic devices or for efficient solar energy conversion. A prominent example is given by the repeated and reversible switching of micro-patterned phase-change materials, typically chalcogenide alloys, that are used in commercial data storage media such as compact disks.^2^ Under a weak and short pulse of infrared light (heat), these materials quickly morph between the crystalline and the amorphous phase accompanied by a pronounced optical reflectivity contrast of the two structurally distinct phases.^3^

In view of these applications, spin-crossover (SCO) nanomaterials constitute a particularly interesting class of phase-transition materials, that can be switched between a low-spin (LS) state and a high-spin (HS) state, involving the rearrangement of electrons in the *t*_2__*g*_ and *e_g_* orbitals of *d*^4^–*d*^7^ transition metal atoms.^4–7^ The SCO process can be triggered by variations in temperature, pressure, magnetic field, or light irradiation,^8^ and it is accompanied by pronounced changes in structure and color. The thermal LS → HS transition is an entropy-driven endothermic process, in which the major entropy gain contribution originates from the increased density of vibrational states in the HS state due to the weakened metal-ligand coordination bonds; a smaller (∼25%) contribution arises from the increase in spin degrees of freedom.^6^ In view of data storage and optoelectronic applications, SCO nanomaterials have recently been synthesized as either nanoparticle dispersions or nanometric thin films,^9–12^ but many open questions remain regarding size effects and cooperativity at the nanoscale.^13,14^

Besides many steady-state studies,^7^ impulses of external stimuli, such as temperature jump, magnetic field pulse, and pressure jump, have been employed to investigate nonequilibrium SCO dynamics.^6^ Moreover, short pulses of visible light can populate the HS state via excitation into the metal-ligand charge transfer (MLCT) band, generating nonequilibrium spin fractions with long life times at low temperatures, which was termed light-induced excited spin-state trapping (LIESST).^15^ Light-induced SCO dynamics on diluted solutions,^16,17^ bulk crystals,^18,19^ and nanoparticle ensembles^13,20,21^ have been investigated with femtosecond-microsecond resolution using ultrafast optical and X-ray techniques.

In a previous publication,^22^ we have demonstrated, using ultrafast electron microscopy (UEM), the laser-induced reversible phase transition of individual, isolated SCO nanoparticles lying on a graphite substrate. Nanoparticles of the 3D molecular framework material Fe(pyrazine)Pt(CN)_4_ (Refs. 12 and 23–27) were thermally excited using nanosecond laser pulses at 532 nm, inducing nonequilibrium LS and HS fractions within 10 ns; the nonequilibrium HS fraction partially decays on a 100–300 ns time scale. Single-nanoparticle electron diffraction and real-space imaging were used to follow the unit cell expansion/contraction, and morphology changes accompanying the SCO.

The aim of this contribution is to elucidate the mechanism and time scales of thermally induced non-equilibrium phase transitions, in particular, spin transitions, at the nanometric time and length scales of individual nanoparticles. First, we formulate the differential equations for coupled reaction kinetics and heat transfer including the underlying substrate. Then, we investigate and discuss the effect of various experimental parameters on the simulated SCO dynamics of the Fe(pyrazine)Pt(CN)_4_/graphite system. Finally, we compare the simulated and experimental electron diffraction time profiles, demonstrating that the proposed non-equilibrium phase transition model of Ref. 22 can fully account for the observed switching dynamics.

## II. EXPERIMENTAL

Overviews of the concept of UEM and apparatus are detailed elsewhere.^28^ Briefly, we employ 266 nm, ∼10 ns laser pulses to generate electron pulses via photoemission from a 16 *μ*m LaB_6_ flat cathode incorporated in an FEG gun assembly, in a 200 keV transmission electron microscope. The timing (repetition rate and delay) of the 532 nm optical pump and electron probe pulses is controlled by a digital delay generator. The laser is guided onto the sample by a mirror inside the microscope column with an angle of ∼5° with respect to the incoming electron beam, such that the laser polarization is approximately parallel to the sample plane. The power and pointing stability of the laser beam were monitored *in situ* by a beam profiler camera located at an equivalent image plane of the specimen.^29^ Slight laser beam drifts between measurements were corrected for. Since the nanoparticles are much smaller than the laser beam, the laser fluences reported in this work are the local fluences at the site of the nanoparticle, which corresponds to the center of the Gaussian laser profile.

**FIG. 1.**
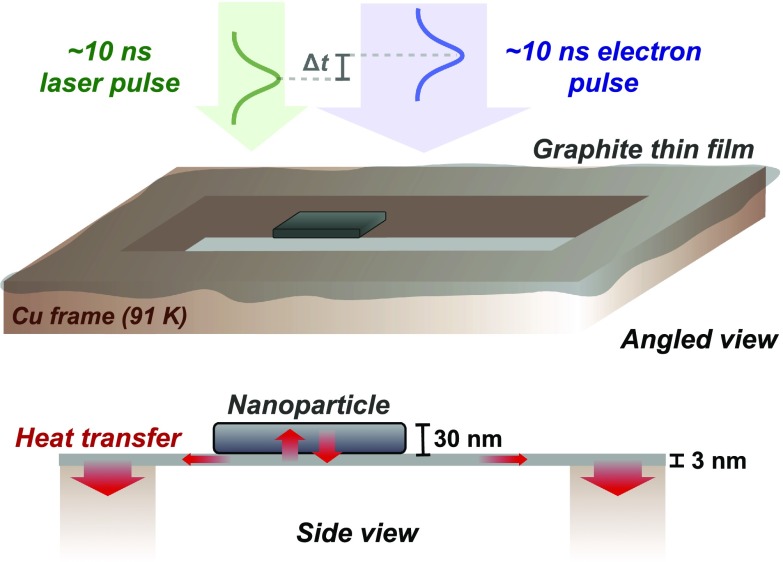
Schematics of the experimental configuration (not to scale). The spin-crossover nanoparticle is situated on a thin graphite film which is supported by a thick copper frame (not drawn to scale) at 91 K. Laser and electron pulses of ∼10 ns are overlapped and synchronized at repetition rates of 600 Hz to 3 kHz. The heat transfer pathways after laser excitation are schematically shown in the bottom side-view panel.

The nanoparticles (30–50 nm thickness) are lying on a thin ∼3 nm graphite substrate (Graphene Supermarket^30^), which is supported by a thick copper frame with 7.5 × 7.5 *μ*m opening, shown in Fig. 1. Single nanoparticles were selected using a selected-area (SA) aperture placed in the image plane of the objective lens. The alignment of the SA aperture was verified and corrected for by comparing the selected area in the image with the shadow image of the defocused direct beam in diffraction. The camera length and the ellipticity in the measurement of diffraction patterns were calibrated and corrected for, respectively, using a polycrystalline aluminum film as a reference.

The sample was carried in a liquid-nitrogen cryoholder and kept at 91 K. To measure the temperature-dependent phase transition curves, the temperature was varied with a rate of ∼2 to 4 K per minute, which should be slow enough to assure thermodynamic equilibrium at the sample during the temperature change. The absolute temperature was calibrated using a reference sample.

## III. NONEQUILIBRIUM PHASE TRANSITION MODEL

In general, heat transfer and chemical reaction kinetics are coupled because the reaction rate changes drastically with temperature and the reaction may generate or absorb heat. A coupled heat transfer and the reaction analysis are commonly applied to large-scale industrial processes.^31,32^ However, such studies of phase transitions on rapid time and nanometer length scales have hitherto not been reported. Below, we will formulate the equations that relate the enthalpy change of the SCO reaction to the time-resolved spin-state fraction and heat transfer rates for the three-body system shown in Fig. 1. For comparison with the experiment, diffraction time profiles are obtained by relating the simulated spin-state fractions as a function of time delay to the lattice parameters of the LS and HS crystal structures. We note that we herein employ a quickly converging continuum mechanics based approach to model the phase transition dynamics, as opposed to discrete-particle elastic switching models described in the literature.^13,33,34^

### A. Coupled reaction and heat transfer

In the steady-state regime, where the system is in thermodynamic equilibrium, the relative spin-state populations are determined by the external temperature, pressure, and magnetic field. In the following, we assume that the pressure and magnetic field remain constant, and mass diffusion does not occur. It is noted that the sample in the electron microscope experiences a magnetic field of about 1 T. The phase transition characteristics and temperature are only marginally affected by this magnetic field.^35^

We assume that each spin center in the nanoparticle is independent, i.e., we neglect interactions among them that could lead to cooperativity. This negligence is reasonable, given the fact that Fe(pyrazine)Pt(CN)_4_ is a relatively rigid spin-crossover polymer compared to softer materials that show strong cooperative effects.^13^ The small hysteresis in the phase transition curve (see Sec. III C) is therefore not taken into account in the theoretical treatment.

The degree of SCO reaction, *ξ* ≡ *ξ_h_*, is defined as the fraction of spin centers in the HS state *ξ_h_*, such that the LS fraction is given by *ξ_l_* ≡ 1 − *ξ*. Then we define the total enthalpy of the SCO system as

$$\begin{array}{c} H\left(T;\xi \right)\equiv \xi_{l}H_{l}\left(T\right)+\xi_{h}H_{h}\left(T\right)=H_{l}+\xi \left(H_{h}-H_{l}\right), \end{array}$$ (1)

where *H_l_* and *H_h_* are the enthalpies of the LS and HS states, respectively. The enthalpy increases slowly with temperature, but it changes abruptly during the phase transition (*ξ*  =  0 → 1).

It follows that the heat capacity of the system (at constant pressure) can be obtained by differentiating Eq. (1) with respect to *T* as

$$\begin{array}{c} C_{p}=\xi_{l}\frac{\partial H_{l}}{\partial T}+\xi_{h}\frac{\partial H_{h}}{\partial T}+\frac{\partial \xi_{l}}{\partial T}H_{l}+\frac{\partial \xi_{h}}{\partial T}H_{h}=\left\{\xi_{l}C_{p,l}+\xi_{h}C_{p,h}\right\}+\frac{\partial \xi }{\partial T}\Delta H, \end{array}$$ (2)

where $C_{p,l}=\frac{\partial H_{l}}{\partial T},\;C_{p,h}=\frac{\partial H_{h}}{\partial T}$, and Δ*H *=* H_h_* − *H_l_*. The first two terms represent the population-weighted heat capacity, and the third term corresponds to the system's heat capacity increase during the phase transition from LS to HS (endothermic reaction, which is driven by entropy).

In a non-equilibrium regime, when the temperature is changed more rapidly than the system can reach equilibrium, the spin-state fractions become time-dependent. In this case, the chemical kinetics, i.e., the rates at which the spin conversion proceeds, need to be taken into account. Because the total energy is conserved, the rate of (internal) enthalpy change per unit volume should be equal to the rate of (external) heat flow per unit volume.^31^ The rate of internal enthalpy change can be calculated by differentiating Eq. (1) with respect to time *t*, resulting in

$$\begin{array}{c} \frac{dH}{dt}\equiv \left\{\xi_{l}C_{p,l}+\xi_{h}C_{p,h}\right\}\frac{\partial T}{\partial t}+\Delta H\frac{\partial \xi }{\partial t}=r\left(t\right)+k\nabla^{2}T-h_{c}\Delta T\equiv \frac{dQ}{dt}, \end{array}$$ (3)

where *k* is the thermal conductivity (not to be confused with the kinetic rate constants *k_l_* and *k_h_*), *h_c_* is the heat transfer coefficient describing the heat flow at the interface between the particle and the substrate, and Δ*T* is the temperature difference across the interface. *r*(*t*) describes the temporal profile of heat deposit by the laser excitation pulse. Note that we utilized Eq. (2) and the property of partial derivatives, $\frac{dH}{dt}=\frac{\partial H}{\partial T}\frac{\partial T}{\partial t}+\frac{\partial H}{\partial \xi }\frac{\partial \xi }{\partial t}$. This differential equation clearly separates the chemical dynamics due to SCO (left-hand side) from the thermal dynamics due to excitation and heat diffusion (right-hand side). Heat transfer through radiation can be neglected.

The solution of Eq. (3) requires the knowledge of the spin fractions, *ξ_h_* and *ξ_l_*, and their rate of change, $\frac{\partial \xi }{\partial t}$. We assume that the time-dependent spin-state fractions in the nanoparticle follow the simple reversible first-order kinetics

$$\text{LS}\left(\xi_{l}\right)\overset{k_{l}\left(T\right)}{\underset{k_{h}\left(T\right)}{⇌}}\text{HS}\left(\xi_{h}\right),$$ (4)

where *k_l_* and *k_h_* are the temperature-dependent forward and reverse rate constants, respectively. The rate of fraction change can then be expressed as

$$\frac{d\xi }{dt}=+\frac{d\xi_{h}}{dt}=-\frac{d\xi_{l}}{dt}=k_{l}(T)\xi_{l}-k_{h}(T)\xi_{h}+P\;\xi_{l}\;n(t),$$ (5)

where *n*(*t*) is the photon density and *P* is the yield of the optical LS → HS excitation pathway (see Sec. III B 1). Equations (3) and (5) constitute a set of coupled differential equations for *T* and *ξ*, which are solved numerically.

The temperature dependence of the rate constants can be described by the Eyring-Polanyi equation^36,37^

$$k_{i}\left(T\right)=\frac{k_{B}T}{h}e^{-\Delta G_{i}^{‡}/RT}=\frac{k_{B}T}{h}\text{exp}\;\left[\frac{\Delta S_{i}^{‡}}{R}-\frac{\Delta H_{i}^{‡}}{RT}\right],$$ (6)

where *h* is the Planck constant, *k_B_* is the Boltzmann constant, and *R* is the ideal gas constant. $\Delta H_{i}^{‡},\;\Delta S_{i}^{‡}$, and $\Delta G_{i}^{‡}$ are the standard enthalpy, entropy, and free energy of activation, respectively, from *i *=* l*, *h* to the transition state. Then, the steady-state equilibrium constant, *K*, is related to the rate constants by

$$K=\frac{\xi_{h}\left(t\to \infty \right)}{\xi_{l}\left(t\to \infty \right)}=\frac{k_{l}}{k_{h}},$$ (7)

which results in

$$K=e^{-\Delta G/RT}=\text{exp}\;\left[\frac{\Delta S}{R}-\frac{\Delta H}{RT}\right],$$ (8)

where $\Delta H=\Delta H_{l}^{‡}-\Delta H_{h}^{‡}=H_{h}-H_{l},\;\Delta S=\Delta S_{l}^{‡}-\Delta S_{h}^{‡}=S_{h}-S_{l}$, and Δ*G*  =  Δ*H* – *T*Δ*S* are the changes of enthalpy, entropy, and free energy, respectively, for the SCO transition from the LS to the HS state.

We utilize Eq. (7) and evaluate *k_l_*(*T*) using *k_h_*(*T*) and *K*(*T*) obtained from time-resolved optical and static phase transition measurements, respectively. The obtained rate constants are then inserted in Eq. (5).

### B. Spin-crossover dynamics simulations

#### 1. Excitation pathways

The SCO reaction can be induced by the change of temperature, pressure, magnetic field, or light. Under the present experimental conditions, we only consider two pathways: (i) optical excitation by direct absorption of visible light by the particle; (ii) thermal excitation by heat resulting from the laser illumination of the particle and the substrate. As stated earlier, the sample in an electron microscope is under zero pressure and a constant magnetic field of ∼1 T.

The optical excitation pathway is negligible in our case as explained in the following. Direct electronic photoexcitation into the metal-to-ligand charge-transfer (MLCT) state of the LS state, exciting an electron from the Fe atom to the pyrazine ligand, populates the low-energy HS state in a strongly non-adiabatic relaxation process with a quantum efficiency close to unity.^16,38^ Each absorbed photon therefore leads to the population of one “molecular” HS state. However, due to the nearly perpendicular (∼85°) orientation of the laser polarization with respect to the transition dipole moment of the MLCT transition (along the *c*-axis) and the low laser fluence used (<10 mJ cm^−2^), we expect the direct photoexcitation conversion efficiency to be less than 1% in the present case (assuming absorption coefficient of 10 000 M^−1 ^cm^−1^; see Sec. III C). In time-resolved spectroscopy experiments on nanoparticle ensembles, it was found that a fluence of 25 mJ cm^−2^ is needed to induce more than 20% population change by means of optical excitation (see Sec. S.II.1 of the supplementary material). In the present experiment, we observe excitation yields of nearly 100% for single nanoparticles at much lower fluences (<10 mJ cm^−2^). This implies that we can neglect the direct photoexcitation pathway and the laser excitation yield *P* in Eq. (5) can be set to zero in the simulations. The SCO dynamics in this study are therefore dominated by the thermal excitation pathway mediated by the substrate.

#### 2. Computational procedure

We consider the situation of three objects in thermal contact: the nanoparticle (500 × 500 nm^2^, 30–50 nm thickness), the graphite thin film (7.5 × 7.5 *μ*m^2^, ∼3 nm thickness), and the copper frame surrounding the graphite film, which is assumed to act as a heat sink at constant temperature, 91 K (see Fig. 1). Since both the substrate and the particle are thin, we can ignore the *z* dependence in Eqs. (3) and (5) and reduce it to a 2D (*x*, *y*) problem with the position of the particle relative to the substrate and the copper grid explicitly considered.

Initially, the temperatures of the particle and the substrate are set to the copper frame temperature, 91 K, at *t* ≪ 0. The HS fraction is set to zero at 91 K for a single-shot experiment simulation. For comparison with the stroboscopic experiment, the initial HS fraction due to spin-state trapping was increased correspondingly (see Sec. IV F 1).

We employ a finite-element method and divide the particle and the substrate into 2D mesh points with sizes of 500/*N* nm. The convergence is obtained when *N* > 4. However, the simulation with 500 nm mesh size does not significantly deviate from the converged calculation; we therefore employed *N* = 1 during refining parameters. The final simulations are performed at 100 nm mesh size (*N* = 5). Equations (3) and (5) are numerically solved for *T* and *ξ* at each mesh point using an ordinary differential equation solver, *lsode*, in the GNU Octave package.^39^

The heating rate, *r*(*t*) in Eq. (3), is calculated from the Gaussian intensity profile in space (*x*, *y*) and time and is attenuated in the propagation direction (*z*) in the material. For the nanoparticle, both the phase transition and the heat transfer are included; for the substrate, only heat diffusion is considered.

After numerical integration, the temperature and the fraction are averaged over all mesh points (for example, 5 × 5 when *N* = 5) of the particle. For the substrate, the temperature was averaged over the mesh points beneath the particle.

#### 3. Diffraction time profiles

The expansion/contraction of the lattice accompanying the spin conversion is expected to be very fast, namely, 10–100 ps for nanoscale materials, dictated by the speed of sound (∼1000 ms^−1^). Heat diffusion and SCO are therefore considered to be the rate-determining processes.

For comparison to our diffraction results, we simply assume that the positions of the diffraction peak are a population-weighted mean value of those of the pure HS (*h*) and LS (*l*) states

$$\left\langle q\right\rangle \left(T;\xi \right)\approx \xi_{l}q_{l}\left(T\right)+\xi_{h}q_{h}\left(T\right),$$ (9)

where $q_{i}=\left|\left(h/a_{i},k/b_{i},l/c_{i}\right)\right|$ are the momentum transfer values of a particular diffraction peak (hkl) for a tetragonal lattice (with unit cell parameters *a_i_*, *b_i_*, *c_i_*; *i *=* l*, *h*).^24^ This expression is justified by considering that the electron coherence length in our experiment dictates the rather large diffraction peak width (0.08 nm^−1^). Small width changes or peak splittings due to domain formation are therefore obscured. Furthermore, an isolated HS unit cell embedded in a largely homogeneous LS lattice acts as a lattice defect, distorting the lattice structure and inducing a local lattice expansion, which is proportional to the concentration of HS centers.

The temperature dependence of the crystal structure due to thermal expansion is explicitly considered by assuming

$$d_{i}\left(T\right)=d_{i}\left(T_{c}\right)+\frac{dd_{i}}{dT}\left(T-T_{c}\right),$$ (10)

where *d_i_* (*i *=* l*, *h*) are the unit cell parameters *a_i_*, *b_i_*, *c_i_*, and *T_c_* is the phase transition temperature.

The *ξ*(*t*) and *T*(*t*) profiles from the numerical simulation are then inserted into Eqs. (9) and (10) in order to obtain the momentum transfer profiles ⟨q⟩(t) for direct comparison with the pump-probe electron diffraction experiment (see Sec. IV F).

### C. Nanoparticle parameters

The dimensions of the nanoparticle and the substrate are directly obtained from imaging in the electron microscope. The thickness of the particle is estimated to be ∼50 nm from electron energy loss experiments.

The optical cross section of the nanoparticle is not exactly known. Typical extinction coefficients for MLCT transitions in transition-metal complexes are 10 000 M^−1 ^cm^−1^.^40^ However, because the laser polarization (in the *a*, *b*-plane) is almost perpendicular to the transition dipole moment (aligned along the Fe-pyrazine *c*-axis), the absorption in the present case is expected to be much lower. For the MLCT transition dipole moment rotated 85° with respect to laser polarization, the extinction coefficient becomes ∼76 M^−1 ^cm^−1^. With a heat capacity of 250 JK^−1 ^mol^−1^ for Fe(pyrazine)Pt(CN)_4_,^23^ a laser fluence of 7 mJcm^−2^, and a particle thickness of 50 nm, the direct temperature jump in the particle is therefore only ∼3 K.

The heat capacities of the LS and HS states (250 JK^−1 ^mol^−1^ from Ref. 23) are assumed to be identical, and their temperature dependence is ignored. Neither the heat conductivity nor the heat diffusivity of Fe(pyrazine)Pt(CN)_4_ is known. Here we assume that the diffusivity is 1 × 10^−6^ m^2^ s^−1^ (and the thermal conductivity is 1.11 W K^−1 ^m^−1^), a typical value for these kind of materials.^18^ Since the particle is very thin, thermal equilibration is fast (<1 ns) compared to our temporal resolution (∼15 ns), and therefore the magnitude of the diffusivity does not affect the simulation results. The heat transfer coefficient, *h_c_*, strongly depends on the nature of the interface and is adjusted from particle to particle to match the observation (typically *h_c_* ∼1 × 10^6^ J K^−1 ^m^−2 ^s^−1^). The Biot number for a characteristic length of ∼10 nm becomes 10^−2^, which indicates that heat diffusion is much faster than heat transfer.^41^

**FIG. 2.**
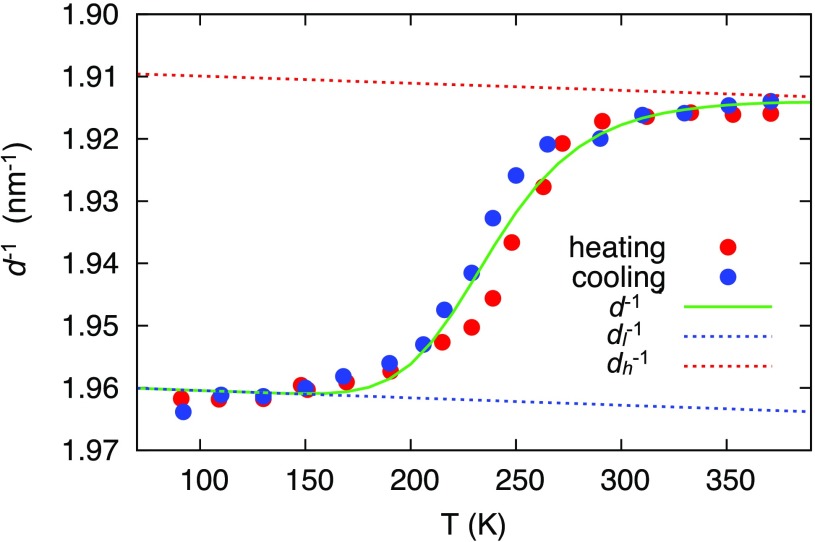
Temperature dependence of the (110) diffraction peak of an ensemble of Fe(pyrazine)Pt(CN)_4_ nanoparticles (experimental error bar = 0.005 nm^−1^). Blue and red circles represent the data during the cooling and heating cycle, respectively. Blue and red dashed lines indicate the temperature dependence of the (110) diffraction peak positions of the LS and HS, respectively, using Eq. (10) only. The slope is related to the negative thermal expansion in the *a, b*-plane of Fe(pyrazine)Pt(CN)_4_ (Ref. 24) (see text). The green solid line is a fit to both heating and cooling curves using Eq. (9).

Due to the limited temperature range of the experimental apparatus, we cannot reliably determine all the parameters (enthalpy and entropy change, lattice parameters, expansion coefficients) simultaneously by fitting Eqs. (7)–(9) to the measured phase transition curves, shown in Fig. 2. We therefore adopted an enthalpy change of Δ*H* = 21.5 kJ mol^−1^ for the polycrystalline Fe(pyrazine)Pt(CN)_4_ compound obtained from differential scanning calorimetry (DSC) measurements (Section S.I in supplementary material), which is in good agreement with the Δ*H* = 21 kJmol^−1^ value reported in the literature.^23^

By using a negative thermal expansion coefficient of $\frac{1}{d_{i}}\frac{dd_{i}}{dT}=-6\times 10^{-6}\;K^{-1}$ for both the LS and HS states (see Sec. IV F 1 below), we then obtained (assuming 0.05 nm^−1^ error in diffraction peak positions) Δ*S* = 87 ± 17 JK^−1 ^mol^−1^, and $T_{c}=\frac{\Delta H}{\Delta S}=241\pm 47\;K$, both in good agreement with the reported entropy change and phase transition temperature.^23^ In addition, the *a,b*-lattice constants of the pure LS and HS structures are fitted to *a_l_* = *b_l_* = 7.21 ± 0.05 Å and *a_h_* = *b_h_* = 7.40 ± 0.07 Å at *T_c_* (*a_l_* → 7.22 Å at 90 K and *a_h_* → 7.39 Å at 400 K), also in reasonable agreement with reported values.^24^

The solution of Eqs. (3) and (5) requires the knowledge of *k_h_*(*T*) and *k_l_*(*T*), i.e., the forward and reverse rate constants for the LS/HS conversion. The relaxation times of the optically induced HS state to the LS state were measured spectroscopically as a function of temperature below the phase transition. These data (see Fig. S3 in the supplementary material) were fitted to the Eyring-Polanyi equation, Eq. (6), in the temperature range 100–160 K, and then extrapolated to higher temperatures. The satisfactory agreement with the data indicates that tunneling does not play a significant role in the back-relaxation for this temperature range. The fitted enthalpy and entropy of activation are in $\Delta H_{h}^{‡}=11\pm 2\;{\text{kJmol}}^{-1}$ and $\Delta S_{h}^{‡}=-79\pm 10\;{\text{JK}}^{-1}{\text{mol}}^{-1}$. The forward reaction constant, *k_l_*(*T*), is then calculated from *K* using Eqs. (7) and (8). As we will show below, the rate constants are slightly adjusted in the simulation in order to match the experiment (see Sec. IV F 1).

### D. Substrate parameters

The thickness of the graphite substrate was estimated to be ∼3 nm from the electron energy-loss spectrum and the inelastic mean free path of ∼160 nm for carbon.^42^ It is noted that for such thin substrates, the etalon effect^43^ plays a role in light absorption due to the constructive interference between transmitted incident light and (coherent) multiple internal reflections within the film. Namely, a thin substrate becomes a resonator cavity where light with a wavelength much longer than the thickness closely satisfies resonance condition, i.e., the propagation inside experiences almost no phase change, and the internally reflected light is almost in phase with the transmitted light at the incident surface of etalon. This results in vanishing reflection and increased absorption in thin graphite or graphene films.^44^ The formula for the etalon^45^ predicts that 21% of light at 532 nm would be absorbed by 3 nm (10 layers) graphite ($\tilde{n}=2.72+1.56i$) film, which is in accord with 2% absorption by a single layer graphene ($\tilde{n}=2.68+1.22i$).

**FIG. 3.**
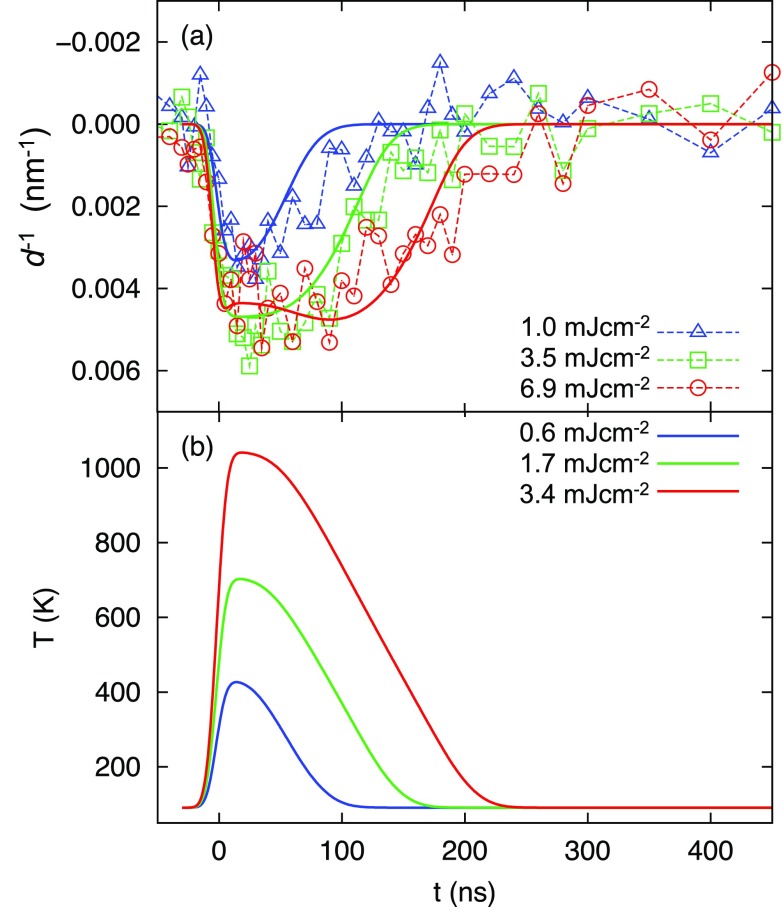
Graphite substrate dynamics. (a) Relative peak shifts of the (220) reflection for three different laser fluences (triangles, squares, and circles) and simulated diffraction profiles as described in the text (solid lines). The data were obtained by measuring the diffraction in the middle of a 7.5 × 7.5 *μ*m^2^ graphite film, framed by a copper grid at 91 K. (b) Simulated temperature profiles of the bare graphite substrate at three different laser fluences.

The thermal properties of graphite strongly depend on temperature. In addition, depending on the quality and type of the graphite film, they can differ as much as several orders of magnitudes.^46^ The properties of the CVD graphite film employed in the present experiment^30^ are not exactly known. We therefore measured its thermal expansion response upon laser-induced *T*-jump excitation, shown in Fig. 3(a) for three excitation fluences. The dynamics were recorded in the middle of a 7.5 × 7.5 *μ*m^2^ graphite film, framed by a copper grid at 91 K. It is noted that the relative momentum transfer value of the (220) reflection increases upon excitation. This implies a contraction of the lattice in the *a*,*b*-plane, in accordance with the well-known negative thermal expansion of graphite in the temperature range of 10–500 K.^47^

Using the temperature-dependent thermal expansion,^47^ thermal conductivity,^46^ and heat capacity^48^ of pyrolytic graphite, the time-resolved behavior in Fig. 3(a) could not be simulated. Pyrolytic graphite is an excellent heat conductor, and its time scale for thermal relaxation is less than 10 ns, i.e., much faster than what is experimentally observed. The contraction of the substrate observed here is larger than the contraction expected for bulk pyrolytic graphite under similar experimental conditions. In order to derive the thermal properties of the graphite film in the present experiment, we therefore scaled the thermal conductivity using

$$k{\left(T\right)}^{-1}\equiv k_{g}{\left(T\right)}^{-1}+k_{c}^{-1},$$ (11)

where *k_g_* is the heat conductivity of pyrolytic graphite as a function of temperature,^46^ and *k_c_* = 62 WK^−1^ m^−1^ is a constant that is determined by optimizing the agreement between the simulated and experimental diffraction time profiles, shown as solid lines in Fig. 3(a). The resulting heat conductivity (see Fig. S4 of supplementary material) is roughly two orders of magnitude smaller than that of pyrolytic graphite, which can be attributed to an increased resistance due to impurities, defects, and the flakiness of the commercial CVD graphite film.

The temperature-dependent heat capacity of graphite is taken from Ref. 48 without modification. Between 90 and 1000 K, *C_p_* of pyrolytic graphite ranges from 1.5 to 20 JK^−1^ mol^−1^, i.e., more than a factor 10 increase. This largely affects the time-dependent temperature profile after photoexcitation, as shown in Fig. 3(b) (using the modified conductivity parameter discussed above). Both the heat capacity and thermal diffusivity decrease above 91 K, resulting in a saturation-like behavior for high laser fluences, and a non-exponential (rather linear) temperature decay profile. We note that, in order to obtain satisfactory agreement between simulation and experiment, the laser fluences used in the simulation are about a factor of two smaller than the experimental fluences. We ascribe this discrepancy to uncertainties in measuring the experimental beam profile and spot size in the exact sample plane of the electron microscope. However, this discrepancy does not affect the conclusions drawn. All laser fluences mentioned in the text below refer to simulation fluences, unless otherwise noted.

The change of lattice parameter of the graphite substrate was modeled as a mixture of graphite^47^ (92%) and graphene^49^ (8%), the latter taking into account the extreme thinness of the CVD graphite film. Graphene exhibits a higher negative thermal expansion over an extended temperature range,^49^ the admixture of which results in a better agreement with our data. The resulting modified thermal expansion properties of graphite are plotted in Fig. S4 of the supplementary material.

## IV. RESULTS AND DISCUSSION

### A. Nonequilibrium SCO dynamics in the phase diagram

**FIG. 4.**
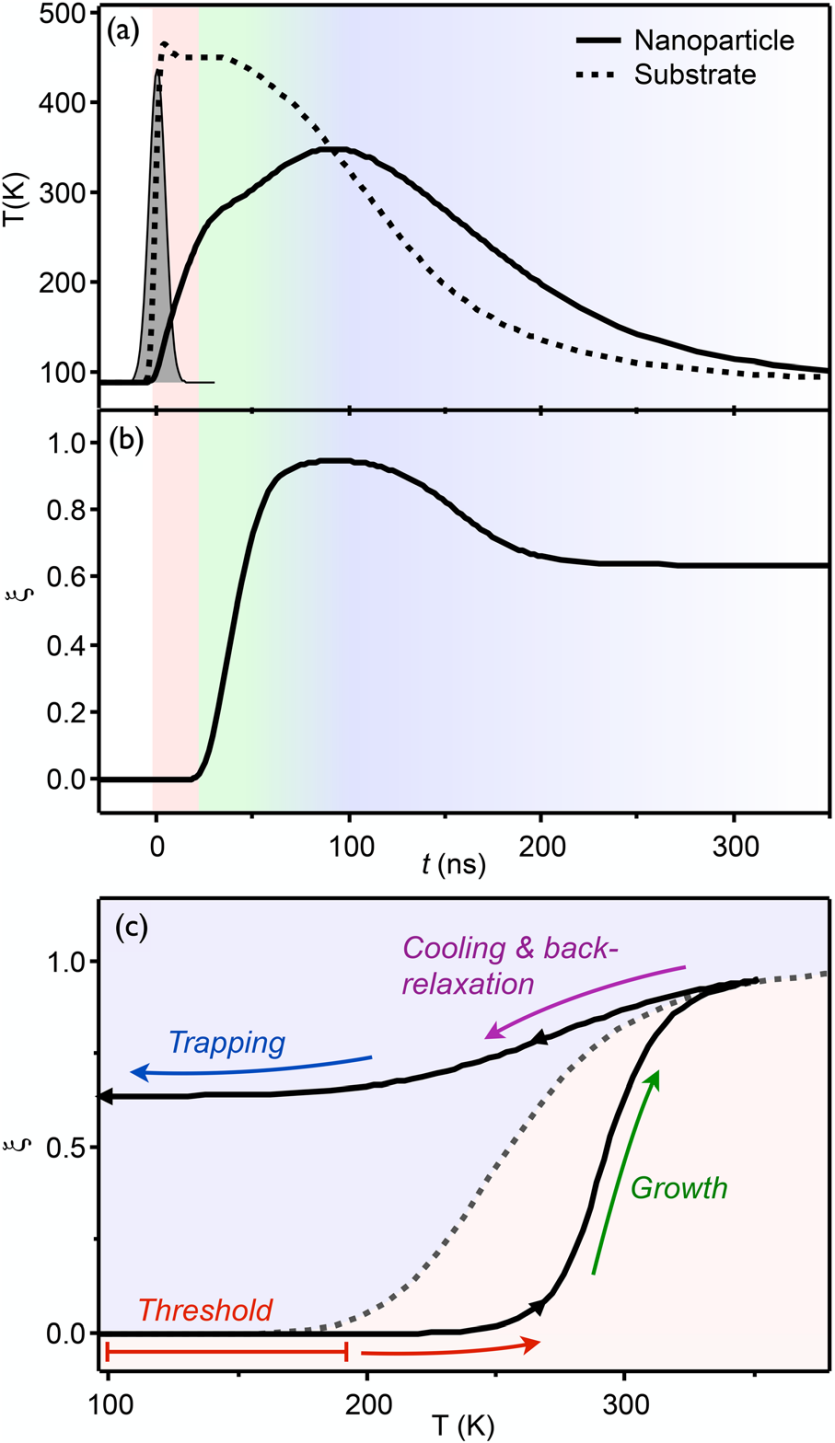
Spin-crossover dynamics. (a) Time-dependent temperature profiles for the graphite substrate (underneath the particle) and the nanoparticle. The laser intensity profile is shown as a Gaussian around *t* = 0 (arbitrarily scaled). (b) Temporal evolution of the HS fraction. Note the ∼20 ns delay in the onset of HS growth. (c) Simulated temporal evolution of the temperature and HS fraction in the SCO phase diagram of the nanoparticle (black line). The equilibrium phase transition is denoted by a dashed line. The various switching processes of heating (red), HS growth (green), and cooling/trapping (blue/purple) are color-coded in panels (a) and (b). The laser fluence in the simulation is set to 1.4 mJ cm^−2^, the particle thickness is taken as 50 nm, and the heat transfer coefficient is *h_c_* = 1.1 × 10^6^ J K^−1^ m^−2^ s^−1^.

The results from the simulations described in Sec. III can now be plotted as a function of time delay after excitation, as shown in Fig. 4 for a laser fluence of 1.4 mJ cm^−2^. Figures 4(a) and 4(b) plot the time profiles of the temperatures and the HS fraction. The trajectory in Fig. 4(c) shows the simulated temporal evolution (following the arrows) of the nanoparticle temperature *T*(*t*) and HS fraction *ξ_h_*(*t*) of Figs. 4(a) and 4(b), as obtained from the numerical simulations, which is compared to the phase transition curve under thermodynamic equilibrium conditions (dashed curve).

Initially, the nanoparticle is in the pure LS state at 91 K. Upon laser excitation, the temperature of the graphite substrate rises promptly, reaching ∼450 K at *t* = 4 ns. The temperature in the nanoparticle rises more slowly, dictated by the heat transfer at the interface. When the temperature of the particle exceeds the thermal threshold of ∼200 K, the particle experiences a thermodynamic driving force to increase its HS fraction, which is governed by the equilibrium constant *K* [Eq. (7)]. At early times after exceeding the threshold temperature, the forward rate constant *k_l_* is not sufficiently high (see Fig. S3 of supplementary material), such that the temperature continues to increase without a noticeable increase in HS fraction. When the temperature is high enough such that *k_l_* becomes larger than the rate of heating, the HS fraction starts to grow using the heat that is supplied by the underlying substrate. The temperature continues to increase, but at a slower rate than previously, because the heat is partially utilized for spin crossover. For a laser fluence of mJcm^−2^, a maximum HS fraction of ∼98% is reached at *t* ≃ 100 ns. It is noted that the SCO reaction is significantly delayed with respect to the thermal excitation pulse by a period during which the particle's temperature increases until 250 K, as is clearly seen by comparing the time profiles in Figs. 4(b) and 4(c). This “onset delay” was experimentally observed, as shown later in Sec. IV F.

The graphite substrate cools down by transferring heat to the copper frame. Once the temperature of the graphite becomes lower than that of the nanoparticle, the heat flow across the interface reverses and the particle commences cooling down. The rate of cooling is again determined by the heat transfer at the interface but also by the cooling rate of the substrate itself. At the early stages, the cooling rate competes with the back-relaxation rate *k_h_* for the HS → LS conversion. The latter is quite fast at high temperature (thermally activated process, see Fig. S3 of supplementary material), but it becomes increasingly slower when the particle cools down. Below a certain temperature, the electronic lifetime becomes sufficiently long that a portion of the particle gets trapped in the HS state (*k_h_* < 1.0 × 10^3^ s^−1^). At the equilibrium temperature of 91 K, the lifetime of the spin-forbidden HS → LS relaxation becomes several ms.

The simulations in Secs. IV B–IV E were performed for a nanoparticle of 50 nm thickness, located in the middle of the copper grid, a laser fluence of 1.4 mJ cm^−2^, and a heat transfer coefficient of *h_c_* = 1.1 × 10^6^ J K^−1^ m^−2^ s^−1^, unless noted otherwise.

### B. Fluence dependence

**FIG. 5.**
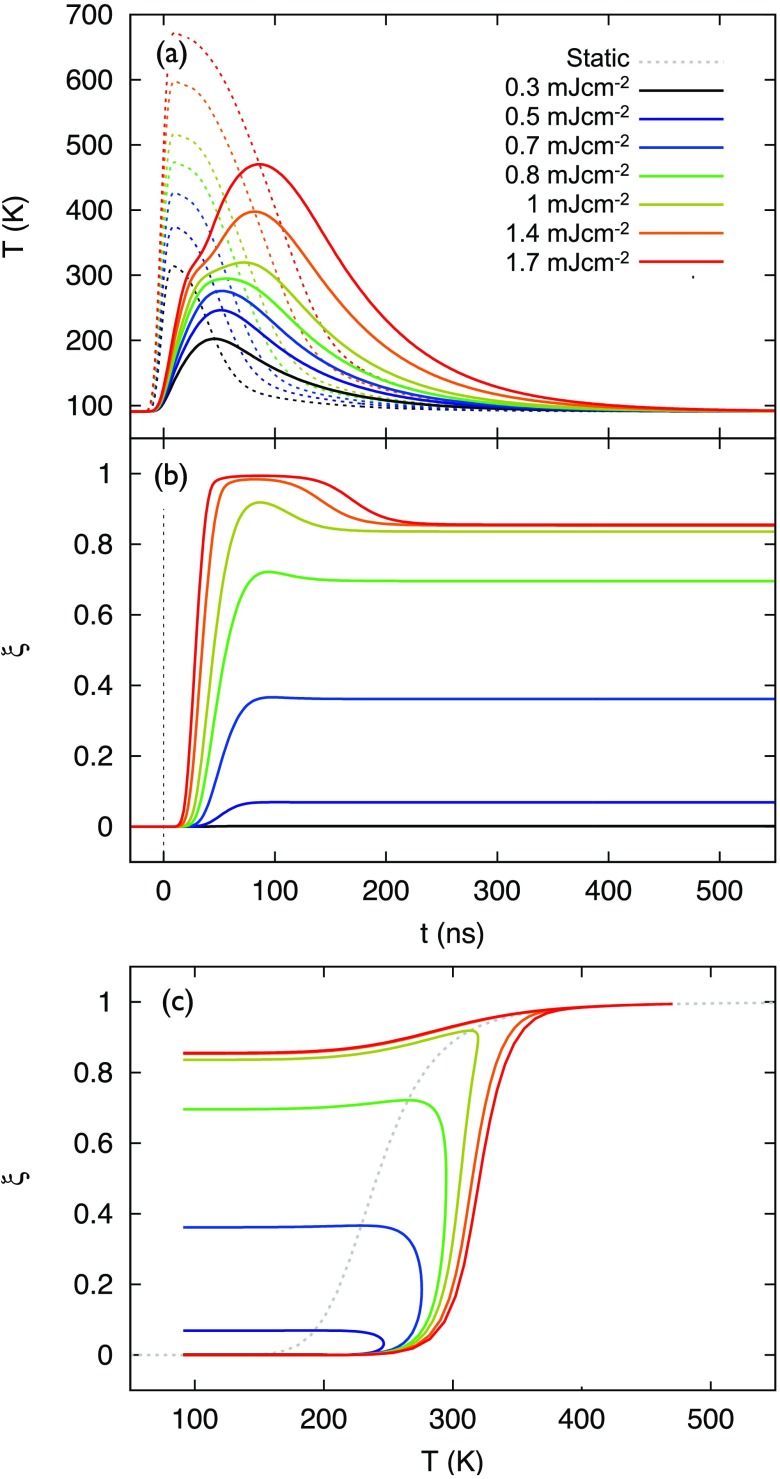
Laser fluence dependence. Simulated temporal evolutions of (a) nanoparticle temperature (solid lines) and substrate temperature (dashed lines) as a function of laser fluence, and (b) corresponding HS fraction time profiles. (c) Temporal evolution of the temperature and HS fraction in the SCO phase diagram for different laser fluences [indicated and color coded in panel (a)]. The dashed line is the equilibrium phase transition curve.

Figure 5 shows the simulated SCO time profiles as a function of the laser fluence. At very low fluence (0.3 mJ cm^−2^), the maximum temperature in the nanoparticle reaches ∼200 K, which is not sufficient to overcome the thermal threshold for SCO; i.e., the nanoparticle remains in the LS state. The temperature rise for a fluence of 0.5 mJcm^−2^ slightly exceeds the threshold. It reaches ∼246 K at which the steady state fraction is as high as 48%. However, the forward reaction rate *k_l_* is still rather low at ∼246 K (see Fig. S3 of supplementary material) and the particle cools down quickly due to the short thermal relaxation time of graphite at low excitation fluence [Fig. 3(b)]. The HS fraction therefore only reaches 7%, which almost entirely gets trapped at longer delay times.

**FIG. 6.**
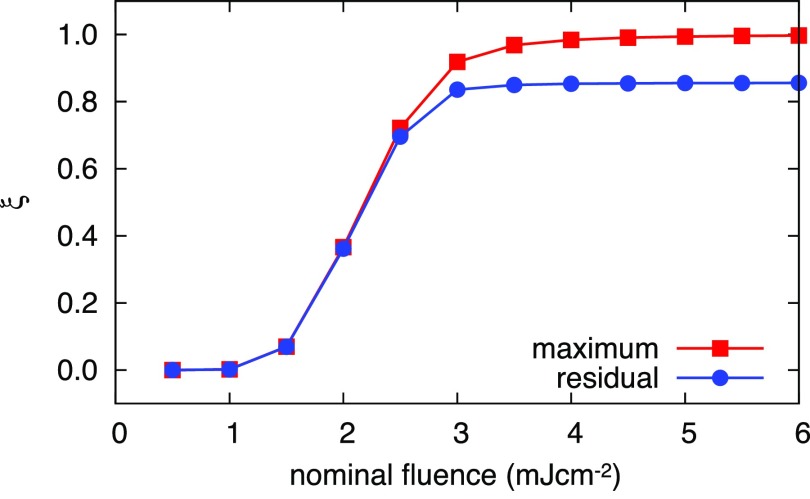
Fluence dependence of the maximum (red) and the residual (blue) HS fractions at 1 *μ*s after excitation.

At intermediate fluences (0.7–1.4 mJ cm^−2^), the temperature of the nanoparticle reaches the phase transition region where the heat can be efficiently used for SCO (the enthalpy increases). The forward and reverse reaction rates are higher, and graphite cooling becomes slower in this fluence regime [Fig. 3(b)]. The maximum HS fraction therefore sensitively increases as a function of fluence, and the residual (trapped) HS fraction starts to deviate progressively from the maximum fraction, as demonstrated in Fig. 6. The fine balance between the cooling rate and the HS → LS back-relaxation rate determines the level of trapped HS species.

For high laser fluences (>1.4 mJ cm^−2^), the phase transition proceeds completely. During the cooling process, the reverse relaxation constant *k_h_* is sufficiently high that the HS fraction initially follows the equilibrium phase transition curve. The dynamics of the HS fraction after traversing the phase transition temperature *T_c_* = 250 K becomes identical for all fluences; the onset of the decay is merely delayed for higher excitation fluences. In addition, the amount of trapped HS fraction becomes independent of laser fluence, as seen in Fig. 6. Above 1.4 mJ cm^−2^, the HS fraction clearly saturates, which is manifested as a plateau at 50–100 ns.

### C. Dependence on the interfacial contact

The properties of the contact at the substrate/particle interface differ from particle to particle, depending on the orientation of the nanoparticle with respect to the substrate, the surface quality of the substrate underneath the particle, or the possible attachment of ligands on the nanoparticle (remnants of the synthesis using surfactants). These factors are difficult to control experimentally, and the thermal properties of the interface contact are therefore unknown *a priori* and need to be determined by comparing simulations with measurements.

**FIG. 7.**
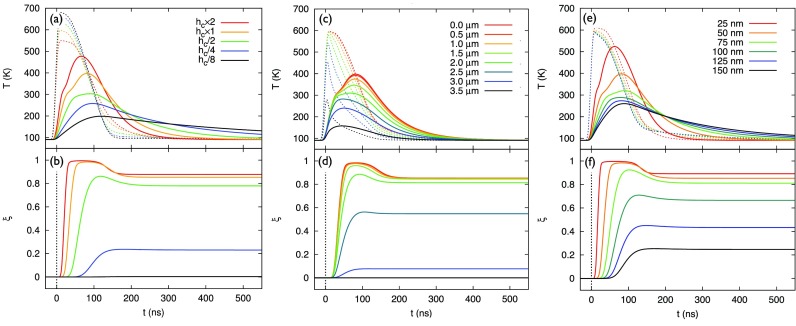
Interface contact, particle position, and particle thickness dependences. Simulated temporal evolutions of (a) nanoparticle temperature (solid lines) and substrate temperature (dashed lines) for different values of the heat transfer coefficient *h_c_* (*h_c_* × 1 corresponds to 1.1 × 10^6^ J K^−1^ m^−2^ s^−1^); (b) corresponding HS fraction time profiles as a function of *h_c_*. Simulated temporal evolutions of (c) nanoparticle temperature (solid lines) and substrate temperature (dashed lines), and (d) HS fraction as a function of particle position for a (circular) graphite substrate (3.5 *μ*m radius) and a laser fluence of 1.4 mJ cm^−2^. A position of 0.0 *μ*m denotes the center of the substrate. (e) and (f) Simulated temporal evolutions of (e) nanoparticle temperature (solid lines) and substrate temperature (dashed lines) for different nanoparticle thicknesses; (f) corresponding HS fraction time profiles.

Figures 7(a) and 7(b) show the simulated dependence of the SCO dynamics and temperature profiles on the interface heat transfer coefficient *h_c_*, for a laser fluence of 1.2 mJ cm^−2^. The higher the value of *h_c_*, the better the thermal contact between the graphite substrate and the nanoparticle.

The trends in dynamics in Figs. 7(a) and 7(b) are distinctly different from those for the fluence dependence in Fig. 5. First, the thermal contact directly affects the rise time of the temperature increase in the nanoparticle [Fig. 7(a)]. The poorer the contact, the slower the temperature rise, and the smaller the maximum temperature jump and, thus, HS fraction [Fig. 7(b)]. Second, due to the longer rise time for poor thermal contact, the thermal threshold is reached at a later time delay, which results in a prolonged onset delay for HS growth. For a thermal contact of *h_c_* = 2.75 × 10^5^ J K^−1^  m^−2^  s^−1^, the rise in the HS fraction begins as late as ∼75 ns after laser excitation. Finally, a poor thermal contact prolongs the cooling time of the nanoparticle, and inversely, the substrate temperature relaxes faster because the heat flow from the particle is reduced.

### D. The effect of thermal inertia

Phase-switching in nanoscale objects is expected to be faster and more efficient than in the bulk, due to their small dimensions and therefore reduced thermal inertia. Thermal inertia is a measure of the response time of the temperature of an object on the transfer of heat to or from the object. Figures 7(e) and 7(f) show the simulated SCO dynamics and temperatures as a function of the thickness of the nanoparticle. The thicker the particle, the larger its thermal inertia.

The thickness dependence shows similar trends as for the thermal contact dependence in Figs. 7(a) and 7(b). For the same amount of heat that flows through the interface, the temperature rise of a thicker particle is protracted and the overall temperature jump that is achieved is smaller. The longer temperature rise time results in a prolonged onset delay for thermal SCO. Furthermore, the decay time for partial LS recovery scales with the particle thickness, and consequently, the residual fraction reciprocally scales with the thickness.

It is concluded that the switching times in the nanoparticles with 30–150 nm thickness range from 50 to 200 ns (including the onset delay), which is still much faster than thermal switching times in the bulk (*μ*s to ms),^19^ despite the indirect substrate-induced excitation in the present case.

### E. Particle position dependence

As was shown in Secs. III D and IV C, the nature of the substrate and interface plays a primary role in determining the time scale and magnitudes of thermal switching of nanoscale objects. Similarly important is the geometry of the substrate and the position of the nanoparticle with respect to the substrate and the heat sink (in this case a copper grid). The presence of the latter is very important in stroboscopic measurements in order to assure a well-defined equilibrium temperature and a fast completion of thermal relaxation of the entire system (including the nanoparticle) prior to the next excitation pulse.

The substrate and particle temperature profiles at different positions on the substrate are plotted in Fig. 7(c) for a calculation assuming a circular graphite film of 7.5 *μ*m diameter. It is noted that the profiles are substantially non-exponential; e.g., at a distance of 2 *μ*m from the center the substrate decay is nearly linear. This is due to (i) the balance between heat diffusion towards and away from the probed area; (ii) the strongly non-linear temperature dependence of the thermal properties of graphite (see Sec. S.III of supplementary material). Closer to the copper grid, the temperature starts to decrease earlier in time. Therefore, depending on where the particle is situated on the grid, it will experience a different time-dependent heat profile from the substrate, which affects the switching magnitude and time scales.

The effect of the particle position on the SCO dynamics is demonstrated in Fig. 7(d). For a nanoparticle at the center of the grid, the temperature gradient between the substrate and the particle is prolonged for tens of ns, during which heat can flow towards the particle and SCO can be induced. The overall conversion efficiency is therefore larger. For particles very close to the heat sink, the temperature jump is not sufficient to induce SCO.

It is noted that the presence of nanoparticles in contact with the substrate affects the thermal dynamics of the substrate itself by effectively increasing its heat capacity and acting as a heat reservoir. Although selected-area diffraction, as used in the present experiment, selectively probes the dynamics of a single isolated particle, if the density of neighboring nanoparticles becomes large, their presence influences the dynamics of the substrate and thus the isolated particle. The simulations in the next Sec. IV F, when we make a comparison with the experimental data, do not take into account these neighboring particles.

### F. Comparison with the experiment

In this section, we compare the SCO simulations with the time-resolved electron diffraction data under various experimental conditions (different fluence and repetition rates). As discussed above, the simulations are based on several assumptions and approximations, such as the (thermal) properties of the nanoparticles and graphite substrate, the exclusion of cooperativity and hysteresis, and the omittance of surrounding nanoparticles. The comparison should therefore only be interpreted qualitatively.

#### 1. Fluence dependence and negative thermal expansion

**FIG. 8.**
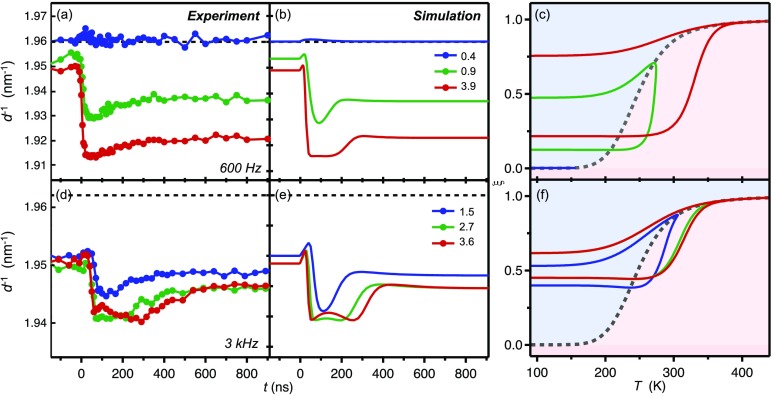
Fluence and repetition rate dependence. (a) Experimental and (b) simulated diffraction dynamics for 600 Hz and three different laser fluences (numbers in mJcm^−2^). Note that the decrease in peak separation in reciprocal space (*d*^−1^) implies an increase in distances *d* in real space. (c) Spin crossover trajectories in the phase diagram corresponding to the simulated time traces of panel (b). (d)–(f) As above, but for 3 kHz and different laser fluences and for a different (similar) nanoparticle. The small decreases around *t* = 0 and the double-peak structure in the 3 kHz traces are due to the negative thermal expansion counteracting the positive expansion accompanying the spin crossover.

In Figs. 8(a) and 8(d), we show the experimental (110) diffraction peak position dynamics for two different laser repetition rates (600 Hz, 3 kHz) and three different laser fluences each. The two data sets were taken on different nanoparticles with similar dimensions. The particle at 600 Hz was located close (∼1 *μ*m) to the copper grid, while the particle at 3 kHz was located nearly in the middle of the grid.

The thermal (volume) expansion coefficient of Fe(pyrazine)Pt(CN)_4_ in the LS state was reported to be negative, $\alpha_{V}=1/V\cdot dV/dT=-2\times 10^{-5}\;K^{-1}$,^24^ causing the unit cell to contract as the temperature increases. Negative thermal expansion behavior in cyanide-bridged compounds is related to the thermal population of low-energy transverse vibrational modes of the cyanide ligands away from the metal-metal axes, with negligible positive expansion of individual bond distances.^50^ This counteracts the positive unit cell expansion affected by the LS → HS SCO. In the simulations, we have taken a linear expansion coefficient in the *a*, *b*-plane of $\alpha_{\text{lin}}=1/l\cdot dl/dT=-6\times 10^{-6}\;K^{-1}$, which is derived by dividing the volume expansion coefficient by a factor of three under the assumption that the thermal expansion is isotropic in the three crystallographic directions. We also assumed that this expansion coefficient is identical for both the HS and LS states.

Signatures of negative expansion (contraction) are visible as small, fast decreases in lattice coordinate around *t* = 0 as shown in Figs. 8(a) and 8(d), and more clearly in the simulations of Figs. 8(b) and 8(e). The period of these features corresponds to the time it takes to exceed the thermal threshold, prior to the increase of the HS fraction [see phase diagrams Figs. 8(c) and 8(f)]. In addition, the contraction is manifested as a double-peak structure around 50–300 ns after excitation in the high-fluence 3 kHz data [Fig. 8(d)], which occurs when the spin transition is saturated.

The simulated profiles qualitatively reproduce the dynamics very well, including the saturation effect and the double-peak features. As expected, the steady-state offset at 3 kHz is larger than the one at 600 Hz, due to the long electronic life time of the HS state at low temperature (several ms), i.e., the period between two consecutive laser pulses is shorter than the relaxation time of the HS state. This is taken into account in the simulation by setting the initial HS fraction to a value that matches the *t* < 0 baseline in the experiment. We do not take into account the possibility of optically exciting the reverse HS → LS state transition as reported by Bousseksou *et al.*,^24^ since absorption cross sections for this excitation channel are expected to be very small.

For the lowest fluence (∼0.4 mJcm^−2^) at 600 Hz, the temperature jump is not sufficiently high to exceed the switching threshold. The particle therefore remains in the LS state, and only a slight contraction is visible around *t* = 0. For higher fluences, the SCO magnitude exhibits a strongly non-linear dependence on the laser fluence (in agreement with the simulations of Fig. 5). The larger the trapped HS fraction, the larger the offset *t* < 0. For the 3 kHz data, saturation (a plateau) is achieved and the trapped HS fraction and offset *t* < 0 become nearly independent of the fluence (see also Fig. 6).

Interestingly, the high-fluence data could only be satisfactorily simulated when the electronic HS → LS back-relaxation rate *k_h_* in the cooling branch of the switching loop was smaller than the one for intermediate fluences. In other words, the higher the initially excited high-spin fraction, the slower the back-relaxation rate at elevated temperatures (∼200 to 250 K) necessary to reproduce the observed decay and trapped HS fraction. We believe that this is a manifestation of hysteresis associated with the cooperativity of the phase transition, which is not explicitly accounted for in the simulation. Indeed, the larger the converted HS fraction, the higher the energy barrier to revert to the structurally different LS structure, the slower the back-relaxation.^51^ However, the present data and the accuracy level of the simulations cannot deduce a more detailed picture of the hysteresis effect on the SCO dynamics. Future experiments at different temperatures and on systems with larger cooperativity are necessary to fully investigate this effect.

It is noted that a satisfactory agreement with the experiment could only be obtained when the SCO “active” fraction in the two nanoparticles was set to 40% and 87% for the 3 kHz and 600 Hz data, respectively. Furthermore, we assumed that the remaining inactive LS fraction is still contributing to the diffraction signal by means of (negative) thermal expansion. The reason for the necessity of this inactive fraction may be twofold: (i) due to surface defects, intra-particle strain, and/or interactions at the substrate/particle interface, a certain fraction of the nanoparticle is inhibited to undergo laser-induced SCO. These SCO centers are excluded from the phase transition dynamics, but may still expand and contract thermally; (ii) a certain fraction of SCO centers underwent a structural transformation over the course of the experiment. The remaining material still displays a thermal expansion behavior. Indeed, it was shown that under certain favorable conditions, the Fe(pyrazine)Pt(CN)_4_ nanoparticles can undergo a chemical transformation that involves the removal of pyrazine molecules from the 3D framework structure.^52^ The remaining crystalline material does no longer display SCO, but it exhibits an exceptionally large negative thermal expansion coefficient in the *a*, *b*-plane.

#### 2. Onset delay

**FIG. 9.**
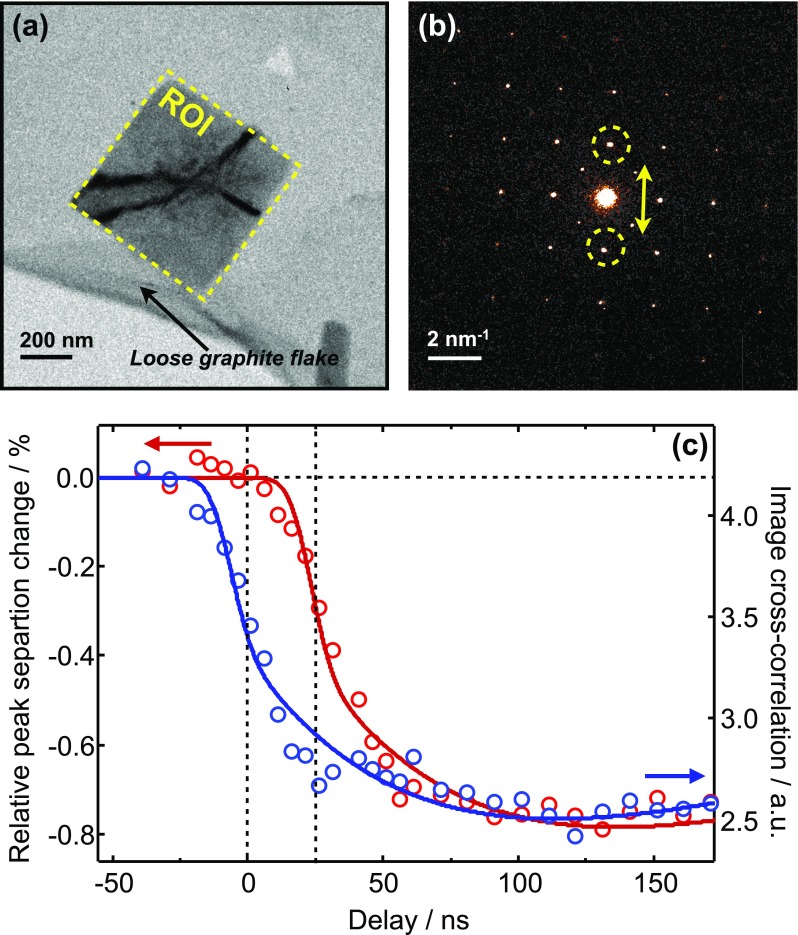
Experimental evidence of the onset delay time. (a) Bright-field image of the nanoparticle lying on a loose flake of graphite substrate. The yellow dashed box indicated the region of interest (ROI) that is used for image cross-correlation; (b) single-nanoparticle diffraction pattern along the [001] zone axis. The yellow circles indicate the (110) diffraction peak pair that is used to follow the diffraction dynamics; (c) comparison of image cross-correlation dynamics (right axis, blue) and diffraction dynamics (left axis, red). The vertical dashed lines denote the approximate delay.

The simulations described in Secs. IV A–IV E showed a pronounced delay of ∼20 ns between the laser excitation and the onset of the HS fraction growth, which was related to the time period that is needed for the particle to heat up to the thermal threshold [cf. Figs. 4(b) and 4(c)]. An experimental verification of this onset delay is shown in Fig. 9.

Figure 9(c) compares the image cross-correlation dynamics^53^ of a region of interest (ROI) shown in Fig. 9(a), with the onset of the diffraction peak position shift indicated in Fig. 9(b). The diffraction dynamics is significantly delayed with respect to the image cross-correlation change. This can be explained by identifying a loose flake of graphite substrate underneath this particular nanoparticle. When the laser excites the sample, the thermal expansion of the substrate causes a tilt of the nanoparticle and a subsequent image contrast change (the dark contours are due to buckling of the particle morphology). This response is almost instantaneous and it therefore gives us a good indication of the “real” *t* = 0 when laser and electron pulses are temporally overlapped. The observed delay of ∼25 ns is in good qualitative agreement with the simulation results. Most other investigated particles lie on a steady graphite film that does not move when laser-heated. In those cases, the image dynamics only relates to the SCO process.

## V. CONCLUSIONS

Studying the factors governing thermal transport at the nanoscale is of importance for many nanotechnology applications. In this contribution, we provide a framework for modeling the heat transport and coupled phase transition dynamics for individual photoswitching nanoparticles lying on a graphite substrate. Upon thermal excitation with a short laser pulse in UEM, the nanoparticles undergo a phase transition from a diamagnetic low-spin state to a paramagnetic high-spin state. We show that the observed switching dynamics and efficiency are sensitively governed by the thermal properties of the substrate, the laser fluence, the interfacial thermal conductance between the nanoparticle and the substrate, and the position of the particle with respect to its supporting framework. With the simultaneous imaging and diffraction capabilities of UEM, in a table-top implementation, we anticipate numerous future applications of heat transport studies for various nanoscale materials and across interfaces, such as single nanotubes, biological fibres, and heterogeneous ensembles of interfacial or embedded structures.

## VI. SUPPLEMENTARY MATERIAL

See supplementary material for differential scanning calorimetry, time-resolved optical experiments on nanoparticle ensembles, and thermal parameters of the graphite substrate.

## References

[1] K. Nasu , *Photoinduced Phase Transitions* ( World Scientific Publishing Co Ptc Ltd., 2004), p. 345.

[2] S. Raoux , Annu. Rev. Mater. Res. 39, 25 (2009). 10.1146/annurev-matsci-082908-145405

[3] A. Sharma , V. V. Tyagi , C. R. Chen , and D. Buddhi , Renewable Sustainable Energy Rev. 13, 318 (2009). 10.1016/j.rser.2007.10.005

[4] *Spin Crossover in Transition Metal Compounds I*, edited by Gütlich P. and Goodwin H. A. ( Springer, Berlin, 2004).

[5] *Spin Crossover in Transition Metal Compounds II*, edited by Gütlich P. and Goodwin H. A. ( Springer, Berlin, 2004).

[6] *Spin Crossover in Transition Metal Compounds III*, edited by Gütlich P. and Goodwin H. A. ( Springer, Berlin, 2004).

[7] M. A. Halcrow , *Spin-Crossover Materials, Properties and Applications* ( John Wiley & Sons, 2013).

[8] P. Gütlich and A. Hauser , Coord. Chem. Rev. 97, 1 (1990). 10.1016/0010-8545(90)80076-6

[9] H. J. Shepherd , G. Molnar , W. Nicolazzi , L. Salmon , and A. Bousseksou , Eur. J. Inorg. Chem. 2013, 653 10.1002/ejic.201201205

[10] S. Cobo , G. Molnar , J. A. Real , and A. Bousseksou , Angew. Chem. Int. Ed. 45, 5786 (2006). 10.1002/anie.200601885 16871608

[11] I. Boldog , A. B. Gaspar , V. Martinez , P. Pardo-Ibanez , V. Ksenofontov , A. Bhattacharjee , P. Guetlich , and J. A. Real , Angew. Chem. Int. Ed. 47, 6433 (2008). 10.1002/anie.200801673 18623300

[12] F. Volatron , L. Catala , E. Riviere , A. Gloter , O. Stephan , and T. Mallah , Inorg. Chem. 47, 6584 (2008). 10.1021/ic800803w 18590329

[13] R. Bertoni , M. Lorenc , H. Cailleau , A. Tissot , J. Laisney , M.-L. Boillot , L. Stoleriu , A. Stancu , C. Enachescu , and E. Collet , Nat. Mater. 15, 606 (2016). 10.1038/nmat4606 27019383

[14] A. Bousseksou , G. Molnar , L. Salmon , and W. Nicolazzi , Chem. Soc. Rev. 40, 3313 (2011). 10.1039/c1cs15042a 21544283

[15] S. Decurtins , P. Gütlich , C. P. Köhler , and H. Spiering , Chem. Phys. Lett. 105, 1 (1984). 10.1016/0009-2614(84)80403-0

[16] C. Bressler , C. Milne , V. T. Pham , A. ElNahhas , R. M. van der Veen , W. Gawelda , S. Johnson , P. Beaud , D. Grolimund , M. Kaiser , C. N. Borca , G. Ingold , R. Abela , and M. Chergui , Science 323, 489 (2009). 10.1126/science.1165733 19074309

[17] W. Gawelda , A. Cannizzo , V. Pham , F. Van Mourik , C. Bressler , and M. Chergui , J. Am. Chem. Soc. 129, 8199 (2007). 10.1021/ja070454x 17559211

[18] M. Lorenc , J. Hebert , N. Moisan , E. Trzop , M. Servol , M. Buron-Le Cointe , H. Cailleau , M. Boillot , E. Pontecorvo , M. Wulff , S. Koshihara , and E. Collet , Phys. Rev. Lett. 103, 028301 (2009). 10.1103/PhysRevLett.103.028301 19659251

[19] M. Lorenc , C. Balde , W. Kaszub , A. Tissot , N. Moisan , M. Servol , M. Buron-Le Cointe , H. Cailleau , P. Chasle , P. Czarnecki , M. L. Boillot , and E. Collet , Phys. Rev. B 85, 054302 (2012). 10.1103/PhysRevB.85.054302

[20] P. Chakraborty , M.-L. Boillot , A. Tissot , and A. Hauser , Angew. Chem. Int. Ed. 52, 7139 (2013) 10.1002/anie.201301562. 23740617

[21] R. Bertoni , M. Lorenc , A. Tissot , M. Servol , M.-L. Boillot , and E. Collet , Angew. Chem. Int. Ed. 51, 7485 (2012). 10.1002/anie.201202215 22696479

[22] R. M. van der Veen , O. H. Kwon , A. Tissot , A. Hauser , and A. H. Zewail , Nat. Chem. 5, 395 (2013). 10.1038/nchem.1622 23609090

[23] V. Niel , J. Martinez-Agudo , M. Munoz , A. Gaspar , and J. Real , Inorg. Chem 40, 3838 (2001). 10.1021/ic010259y 11466039

[24] S. Cobo , D. Ostrovskii , S. Bonhommeau , L. Vendier , G. Molnar , L. Salmon , K. Tanaka , and A. Bousseksou , J. Am. Chem. Soc. 130, 9019 (2008). 10.1021/ja800878f 18570417

[25] Y. Raza , F. Volatron , S. Moldovan , O. Ersen , V. Huc , C. Martini , F. Brisset , A. Gloter , O. Stéphan , A. Bousseksou , L. Catala , and T. Mallah , Chem. Commun. 47, 11501 (2011). 10.1039/c1cc14463d 21935546

[26] E. Collet , L. Henry , L. Piñeiro-Lopez , L. Toupet , and J. Real , Curr. Inorg. Chem. 6, 61 (2016). 10.2174/1877944105666150910233704

[27] M. Castro , O. Roubeau , L. Piñeiro-López , J. A. Real , and J. A. Rodríguez-Velamazán , J. Phys. Chem. C Nanomater. Interfaces 119, 17334 (2015). 10.1021/acs.jpcc.5b05864

[28] A. H. Zewail and J. M. Thomas , *4D Electron Microscopy: Imaging in Space and Time* ( World Scientific Publishing, 2010).

[29] A. H. Zewail and J. S. Baskin , U.S. Patent 0,131,574 A1 (2014).

[30] See https://graphene-supermarket.com/ for (Item number: KU-TEM-CU-2000-025).

[31] C. C. Lee , Commun. Appl. Numer. Methods 5, 539 (1989). 10.1002/cnm.1630050807

[32] E. R. G. Eckert , R. J. Goldstein , W. E. Ibele , S. V. Patankar , T. W. Simon , N. A. Decker , S. L. Girshick , P. J. Strykowski , K. K. Tamma , A. Barcohen , J. V. R. Heberlein , and D. L. Hofeldt , Int. J. Heat Mass Transfer 34, 2931 (1991). 10.1016/0017-9310(91)90070-U

[33] L. Stoleriu , P. Chakraborty , A. Hauser , A. Stancu , and C. Enachescu , Phys. Rev. B 84, 134102 (2011). 10.1103/PhysRevB.84.134102

[34] C. Enachescu , L. Stoleriu , A. Stancu , and A. Hauser , Phys. Rev. Lett. 102, 257204 (2009). 10.1103/PhysRevLett.102.257204 19659117

[35] Y. Ogawa , T. Ishikawa , S. Koshihara , K. Boukheddaden , and F. Varret , Phys. Rev. B 66, 073104 (2002). 10.1103/PhysRevB.66.073104

[36] H. Eyring , J. Chem. Phys. 3, 107 (1935). 10.1063/1.1749604

[37] M. G. Evans and M. Polanyi , Trans. Faraday Soc. 31, 875 (1935). 10.1039/tf9353100875

[38] C. Creutz , M. Chou , and T. L. Netzel , J. Am. Chem. Soc. 102, 1309 (1980). 10.1021/ja00524a014

[39] J. W. Eaton , D. Bateman , and S. Hauberg , *GNU Octave Manual Version 3* ( Network Theory, Ltd., 2002).

[40] I. Krivokapic , P. Chakraborty , R. Bronisz , C. Enachescu , and A. Hauser , Angew. Chem. Int. Ed. 49, 8509 (2010). 10.1002/anie.201004500 20886498

[41] F. P. Incropera , *Fundamentals of Heat and Mass Transfer*, 6th ed ( John Wiley, Hoboken, NJ, 2007).

[42] R. F. Egerton and S. C. Cheng , Ultramicroscopy 21, 231 (1987). 10.1016/0304-3991(87)90148-3

[43] Z. Knittel , *Optics of Thin Films* ( John Wiley Sons Ltd., 1976).

[44] R. R. Nair , P. Blake , A. N. Grigorenko , K. S. Novoselov , T. J. Booth , T. Stauber , N. M. R. Peres , and A. K. Geim , Science 320, 1308 (2008). 10.1126/science.1156965 18388259

[45] V. V. Gozhenko and A. O. Pinchuk , J. Opt. 14, 035705 (2012). 10.1088/2040-8978/14/3/035705

[46] C. Uher , in *SpringerMaterials-the Landolt-Börnstein Database*, edited by Madelung O. and White G. K. ( Springer-Verlag, Berlin, 1991).

[47] W. C. Morgan , Carbon 10, 73 (1972). 10.1016/0008-6223(72)90011-5

[48] H. Shinno , M. Kitajima , and M. Okada , J. Nucl. Mater. 155–157(Pt. 1), 290–294 (1988) 10.1016/0022-3115(88)90256-5.

[49] N. Mounet and N. Marzari , Phys. Rev. B 71, 205214 (2005). 10.1103/PhysRevB.71.205214

[50] A. Goodwin and C. Kepert , Phys. Rev. B 71, 140301 (2005). 10.1103/PhysRevB.71.140301

[51] A. Hauser , Spin Crossover Transition Met. Compd. II 234, 155 (2004). 10.1007/b93641

[52] R. M. van der Veen , A. Tissot , A. Hauser , and A. H. Zewail , Phys. Chem. Chem. Phys. 15, 7831 (2013). 10.1039/c3cp51011e 23598740

[53] H. S. Park , J. S. Baskin , B. Barwick , O.-H. Kwon , and A. H. Zewail , Ultramicroscopy 110, 7 (2009). 10.1016/j.ultramic.2009.08.005 19783100

